# The Foreign Journals: Pathology, Practical Medicine, and Therapeutics

**Published:** 1842-04

**Authors:** 


### PATHOLOGY, PRACTICAL MEDICINE, AND THERAPEUTICS.

### *On Inflammation of the Umbilical Arteries, as a Cause of Trismus Neonatorum.* By Dr. Levy.

This theory was originally promulgated by Colles, in 1818, but has since been opposed by Labatt and Elsässer, whilst it received additional confirmation from the researches of Busch of Berlin, in 1837. Dr. Levy has had more numerous opportunities of investigating this remarkable malady than usually falls to the lot of practitioners in other parts of Europe.

In 1838-9 he attended 22 cases in the Lying-in Institution in Copenhagen. Of these, 20 died and only 2 recovered. Of those that died, 15 were examined carefully after death, and in 14 there were the most decided marks of inflammation in the umbilical arteries. Dr. Levy has found that the principal seat of the inflammatory action is in that part of the umbilical arteries which lies along the urinary bladder. Inflammation is always present in the artery of both sides, though rarely in an equal degree.

Considerable variety was observed in the appearance of the internal coats of the artery, but externally its diameter was always increased, and the peritoneum surrounding it presented evident marks of inflammation; for in several cases this membrane was much injected with red blood, and, in three instances, adhered, by effusions of coagulable lymph, to the jejunum or omentum. On incising the arteries themselves their walls were found to be very much thickened, and their dilated cavities contained more or less of a dark reddish brown or greenish puriform matter, which was always extremely fetid. Upon the removal of this matter the coats of the arteries presented the following alterations:

1. Inflammatory discoloration, and inequality of surface of the tunica intima.
2. Thickening of the tunica intima, or of its subjacent cellular tissue. This spongy tumefaction was one of the most frequent alterations in the umbilical arteries.

3. Ulcerative destruction of the tunica intima, so that in several points this membrane was totally wanting. This appearance was often accompanied by spongy thickening of the subjacent cellular tissue noticed above. These ulcerations were in general superficial, but in two instances the destructive process had extended to the peritoneum. In another case the umbilical artery of the right side was completely perforated, and the spot where this had occurred was surrounded by a layer of thick ichorous matter, which here and there covered also the posterior wall of the bladder.

4. Softening and consequent rupture of the walls of the umbilical arteries. This occurred but once, viz. in that portion of the artery which lies on the superior fundus of the bladder. From this point almost to the navel both arteries were in a state of gelatinous softening, their different coats no longer to be distinguished. At the distance of about an inch from the navel, was an irregularly oval and somewhat lacerated opening in the walls of the artery, from whence about an ounce of ichorous-looking fluid had exuded into the cavity of the pelvis. Even the peritoneum covering the artery was here completely softened, and the whole bore no small resemblance to the well-known gelatinous softening of the coats of the stomach.

5. Gangrene of the umbilical arteries was but once observed; in a child of seven days old, which died forty-eight hours after the commencement of the disease. In the artery of the right side there was found pus, with great thickening of the portion running along the urinary bladder, where also a firm coagulum was discovered attached to the inflamed walls of the vessel. The left umbilical artery was nearly double the size of the right, and externally of a blueish green colour. It contained a large quantity of greenish black ichorous matter, mixed with shreds of the tunica intima, and which exhaled a most putrid and offensive odour.

Dr. Levy paid particular attention in all these cases to the state of the external umbilicus, in order to ascertain how far inflammation of the umbilical arteries can be inferred from the appearance of the navel during life. In four cases it was perfectly unchanged during the whole course of the disease; in ten other instances the surface was in like manner unaltered, but the fundus was more or less red, and filled with a puriform fluid, which quickly reappeared when removed, and in general shortly before death the navel became of a greenish colour.

Dr. Levy then proceeds to examine the theory of Elsässer, who in twenty cases had always discovered after death marks of congestion, and in sixteen of these actual extravasation of blood into the spinal canal. But Dr. Levy believes these appearances to result chiefly from the actual violence necessarily employed in laying open the spinal canal, even in young children; and also perhaps from congestion, as the result of the convulsive disease.

Dr. Levy has twice observed suppuration in the umbilical arteries after death, where no trismus had occurred during life.

He has succeeded in saving two patients by a leech or two applied to the umbilicus, with warm fomentations to the abdomen, and by anti-spasmodics given internally.

*Bibl. for Læger, Sept.* 1840.

---

*An Account of an Epidemic of Typhus, accompanied with Apoplectic and Tetanic Symptoms, which was observed in the district of Cervaro and parts adjacent during the winter and commencement of the spring of* 1840. By Professor de RENZI.

THIS is one of several papers by different physicians on an epidemic which appears to have excited considerable attention. It broke out at Mignano in February, 1840, and caused much alarm from a report which spread among the vulgar that the cholera had made its appearance. This was at once ascertained to be false, and at the end of a month the disease ceased in Mignano, but began to spread at Cervaro and in other communes of that district. Almost all of the inhabitants were more or less affected by the epidemic influence, and suffered from giddiness, lassitude, and great depression of spirits; symptoms which were especially severe in those who had been attacked by intermittents during the previous year.

The disease made its onset differently in different cases. In some persons the first symptom was a sense of formication beginning at the feet and extending over the whole body; others suffered from general uneasiness, pain in the head or back, particularly in the cervical and dorsal regions, attended with difficulty on stooping forward or bending the neck. Occasionally this sensation extended to the lower limbs, and was accompanied with spasmodic contractions. Sometimes the disease was ushered in by an apoplectic seizure with loss of speech and consciousness, lasting for some hours, and followed by a kind of febrile reaction. Other persons fell down in convulsions, with trismus, the neck being drawn forcibly backwards, the whole trunk rigid, spasm of the extremities, and efforts to vomit, sometimes without anything being rejected, while at other times the patient would throw up some lumbrici.

With the exception of a few very mild cases the disease ran much the same course, whatever were the symptoms which ushered in the attack. It might usually be divided into three stages: of which the first was characterized by violent irritation of the nervous centres; the second was the stage of reaction with gastro-nervous fever; the third was attended with putrid or adynamic symptoms. No particular crisis was observed, nor any discharge which could be looked on as critical.

The stage of reaction was accompanied with fever, a hard, quick, and frequent pulse, cephalalgia, and a painful sense of retraction of the head. The pain in the head increased in severity, affecting principally the occiput and extending to the neck and along the spine. In some instances the pain in the spine was dreadfully severe, and the sacrum was referred to as the seat of the greatest suffering. When very violent, this pain was followed by opisthotonos, so complete as to bend the spine into the form of a roman S. Trismus, difficult deglutition, with disinclination for all drinks, subsultus, and a tremulous state of the limbs existed in the severest cases. The tongue was dry, the teeth were coated with sordes, the patient could scarcely speak, the voice was stridulous, except in those instances where there existed complete aphonia. The bowels were costive, and lumbrici were voided by stool, or crept out of the mouth. When the symptoms however presented such severity, the patients usually died in twenty-four or forty-eight hours; or if they survived they continued hemiplegic or lost the use of some of their senses, or fell into a state of low, nervous fever.

In less formidable cases occasional remissions took place, and the nervous symptoms were not so severe, but even then a degree of tetanus and pain in the joints invariably existed. In these cases delirium came on about the fifth day, and was sometimes violent, at other times quiet and muttering. To the delirium, coma succeeded, usually about the seventh day; but when the disease had a fatal issue coma set in very early, even without previous delirium, or after it had existed only for a very few hours. When the patients lived until the second week, the prevailing symptoms were those of adynamic fever, and livid spots frequently appeared on the surface of the body.

General congestion of the intestinal canal, with softening of the mucous membrane of the colon, and injection of the vessels of the peritoneum were found after death. The bowels often contained lumbrici, and the spleen and liver were usually very large. The heart and large vessels contained black and liquid blood; the thoracic viscera generally were much congested, as were the vessels of the brain, but extravasations of blood into the cerebral substance were never met with.

The disease seems to have been very fatal; but Professor Renzi is unable to give statistical details concerning all the communes in which it prevailed. He states, however, that of 218 who were attacked, 116 or more than half died. Those who principally fell victims to the epidemic were young men from sixteen to thirty, especially the robust and healthy. The duration of the disease was very various, some who were attacked dying within a few hours, others in the course of a week, some during the second or third week, and one even so late as fifty days after the seizure. As the epidemic declined, it by degrees assumed the characters of a gastro-rheumatic fever.

Oleaginous purgatives, small doses of calomel, and the solutions of acetate of ammonia and of tartar emetic, were the chief internal remedies in the milder cases; and warm baths, blisters to the extremities, and frictions with belladonna to the spine, the principal external applications. The severer cases were not benefited by any kind of treatment whatever.

Dr. Renzi attributes the disease to the injurious influence exerted on the atmosphere by the cultivation of rice, for though the disease appeared to spread by contagion, yet it did not present such violent symptoms anywhere else as in the immediate vicinity of rice plantations.

*Il Filiatre Sebezio. Luglio,* 1840.

In the same Journal for May, 1841, is a description by Professor Renzi, of a similar epidemic which occurred at S. Marzano, and which, like the epidemic at Mignano, proved very fatal, fifty persons dying out of eighty who were attacked by it. The symptoms so closely resemble those observed at Mignano, that their detail is unnecessary, but the circumstances under which the disease broke out are worth notice. S. Marzano is seated on a level near the Saono, and all the streams from the neighbouring mountains traverse the surrounding country. In January, 1840, the Saono was so swollen by the rains that it overflowed its banks, and, inundating the plain, left the commune of S. Marzano in a sort of island. On the cessation of the rains unusually warm weather set in. The waters rapidly subsided, and the exhalations from the ground became exceedingly offensive. The peasantry, who were predisposed to disease by the privations they had undergone, suffered much from pursuing their labours in this poisonous air, and accordingly in January the disease broke out among them.

The Number for September, 1840, contains an account by Dr. Salvatori of a kindred disease, the typhoid cephalalgia, which was observed among the galley slaves at Procida. This paper affords proof of the contagious nature of the disease, thus confirming the opinion expressed by Professor Renzi. It also shows that when patients are not exposed to the evils of a malarious atmosphere, the disease loses much of its fatal character. At the commencement of April, 1840, some new convicts came to the galleys at Procida, having recently been discharged from the prisons at Potenza, where a disease was then prevailing exactly resembling the epidemic at Mignano. These were the first attacked; those convicts who had been longest employed in the galleys, or such as were inmates of the hospital for some chronic malady suffered next. Other diseases then existing in the hospital soon put on the characters of this typhus, but it did not spread beyond the convict hospital and the galleys. Severe pains in the head and loins occurred as in the epidemic at Mignano. Trismus and coma were not invariably met with, and the whole course of the disease was so mild, that of sixty-two who were attacked by it only one died.

This epidemic in the *circondario* of Cervaro has been made the subject of a special work, as well as of several essays in the Italian journals. Dr. Spada, who was appointed by the Neapolitan government to superintend the treatment of the disease, has written a book upon it, a brief notice of which appears in the Filiatre-Sebezio for December, 1840. This notice contains but little account of the malady beyond what we have already given. It is however interesting, inasmuch as it points out some analogies between the disease and some of those epidemics which a few centuries ago were prevalent throughout Europe. Dr. Spada suggests that there is a similarity between the fever and the acute scurvy of the middle ages, while many of its symptoms resemble those of a convulsive disease to which sheep are subject from eating the poisonous anemone. The reviewer remarks, that its convulsive symptoms bear a closer resemblance to those of ergotism, but does not infer from this that the two diseases are alike in character. He labours indeed on the contrary to prove that the disease which prevailed at Cervaro was nothing else than an epidemic typhus fever. In this opinion we think most will acquiesce who are at all familiar with the descriptions of the malignant fevers which devastated Italy during the 15th, 17th, and early part of the 18th century.

---

*On the Diceras, a rare form of Intestinal Worm.* By Professor ESCHRICHT.

SOME worms were sent to the professor by a friend practising as a physician on the island of Bornholm. They had been passed in enormous quantities by his little daughter after a severe disease, and he was anxious to know their name. To his astonishment, Eschricht recognized in them worms of a kind which Bremser had classed among the pseudhelminthia, and had given a plate of on the title-page of his "Living Worms in Living Men." The only author by whom they were ever described is Sultzer, of Strasburg, the title of whose work is "Diss. sur un ver intestinal nouvellement découvert et decrit sous le nom de Bicorne rude." Strasburg, 1808. His account of their form and structure is most accurate, but Bremser having laid it down that they were nothing but seeds, it has been neglected and almost forgotten.

Eschricht has fully comfirmed Sultzer's description of them, and says that they are veritable entozoa cannot be doubted; but "how it can have happened that notwithstanding the enormous quantity of them found in these two cases, they have occurred only in these two, and in these, once in 1804, at Strasburg, and once in 1841, on the island of Bornholm, is certainly hard to explain, especially to me and others who do not believe in equivocal generation."

*Müller's Archiv.* Heft v. 1841.

---

*Disease of Sappers and Miners.* By Dr. KANZLER, of Mainz.

DURING the exercises of the 7th and 8th pioneer-divisions last August, at Coblentz, I had frequent opportunities of observing this peculiar (and as far as I know undescribed) disease, in all its forms. When the mines laid in the long subterranean passages, called galleries, were fired by trains and blown up, the air in the galleries was at once purified by appropriate machines; but a complete purification of it was quite impossible, and very often the miners would go into it too soon. It was therefore not uncommon to see within five minutes after an explosion, fifteen or twenty men brought out attacked with the *mine disease.* In this condition I saw altogether upwards of 200.

The disease presented itself in different degrees. In the slightest degree there was only a heavy pain in the head, situated especially at the forehead and temples, and giving notice to those who were affected with it, that they should leave the galleries and go into the open air; which if they did not do, a higher degree of the affection soon came on. In the fresh air the headach commonly soon ceased; but it usually came on again as soon as the patients went back into the galleries, and it thus rendered them unfit for work for the whole day.

In a severer form of the disease the patients were seized with an excessive oppression at the chest, as if it were tied round with a cord; an instant after they lost their consciousness, fell to the ground, and had to be carried out of the gallery. Sometimes a third and higher form occurred, in which to the symptoms just described there were added clonic spasms, the patient's face becoming pale or livid, foam appearing at the mouth, and the thumbs being firmly bent in upon the palms. There was also a peculiar form of the disease in which the patients fell into a kind of drunkenness; they grew very talkative at their work, laughed incessantly and without cause, and jumped and danced about just like tipsy men. This state in some cases ceased on exposure to the fresh air; but in others it suddenly gave place to the severest headach, and before they were seen the patients fell down unconscious and convulsed. The unconsciousness and convulsions sometimes lasted for but a few minutes, sometimes for half an hour; and they were almost always followed by the condition which the patients described as the most painful of all, namely, a constant retching and a most terrible nausea, which only rarely ended in actual vomiting. At the end of all the disease a distressing heavy headach came on, and in some cases lasted three days.

The cause of this affection was of course the inhaling of gases which were formed in the galleries by the explosion of the powder. The form which presented symptoms like those of drunkenness was probably due to the production of nitrous oxyde, which, it is well-known, when inspired in sufficient quantity has intoxicating properties.

The treatment was very simple. If there were nothing but headach, the greatest relief was afforded by pouring some vinegar on a handkerchief and making the patient inhale its vapour; and with this fastened under his nose he might even go back into the first part of the gallery and continue his work. When men were brought out unconscious, dashing them with cold water, and the snuffing of strong odours were employed; and when the nausea and retching came on, nothing acted more quickly or certainly than a spoonful of wine with 20 or 30 drops of spts. æther. sulph. In some cases of severe convulsions, when the face was dark, livid, and the pulse full and large, bleeding was beneficial. In those who had the drunken form of the disease, it answered best to let them recover slowly in the fresh air, and then to make them keep quiet.

*Casper's Wochenschrift. Juli* 17, 1841.

---

### *On the Contagious Nature of Pemphigus Neonatorum.*
### By Dr. Scharlau, of Stettin.

[The following observations seem to prove that popular opinion, which has regarded this disease as contagious, is more correct than that commonly entertained in the profession. Judging by the character of this and many others of his papers that have fallen under our notice, the author is one on whose statements full reliance may be placed.]

A well-conditioned child, born of healthy parents, was attacked on the fourth day after birth with an eruption of bullæ varying in size from that of a lentil to that of a hazel-nut, and increasing daily in number. The fluid which they contained was yellowish-white, strongly alkaline, and mixed with a number of oval and round corpuscles, visible only with the microscope. The eruption took place at different periods, so that many of the bullæ were dry when others first broke out.

A second perfectly healthy child was bathed in the same water and washed with the same sponge as the preceding; and after a few days an eruption, coming out in pustules and drying in scales, appeared on its head, and at the same time one of bullæ exactly like those on the infant came out on the neck and legs. The mother, the nurse who bathed the child, and a girl twelve years old who had been with them both, were also at the same time affected with an eruption of bullæ on the hands, face, and legs in distinct spots. The author himself, who had often handled the infant, and then put his hand upon his chin and his lower jaw, soon after felt a burning pain there, as if it had been rubbed with caustic, and this was followed by the appearance of a red spot with a yellow pustule, which on disappearing left a small scurfy patch.

A small quantity of the fluid from a bulla was inserted under the cuticle of the fore-finger. After some time a burning sensation like that caused by nettles was felt, and after thirty-six hours an irritating pain, with a rosy red patch with a dark spot in the middle. Forty-eight hours after the inoculation, the pain had increased, and the cuticle was elevated in little papillæ like grains of sand; sixty hours after, a bulla containing a clear lymph was fully developed, and this, after seventy-two hours, acquired a yellow colour, and then formed a crust.

Three pustules on the neck of the second child were touched with concentrated acetic acid thirty-six hours after the formation of the red spot and the elevation of its centre. After twelve hours the redness had diffused itself more widely, but after twenty-four it completely vanished without any formation of bullæ.

Ordinary baths appearing of no benefit, and internal medicines producing no good effect, the author was induced, by the result of the experiment just mentioned, to add to each bath three ounces of common vinegar. The result was very striking; for the eruption, which had gone on increasing for fourteen days, was now in a few more completely cured. The author supposes that this was brought about by neutralizing the alkaline fluid of the bullæ, and suggests that in the cases, such as are recorded, in which the fluid is acid, alkalies should for the same purpose be added to the baths.

*Casper's Wochenschrift. Marz* 20, 1841.

---

*Account of an Epidemic of Sweating Sickness, which prevailed in several Communes of La Dordogne and La Charente.* By MM. RAYER and BRICHETEAU.

THE epidemic broke out in May, 1841, the weather being at that time very hot, southerly winds prevalent, and the atmosphere loaded with electricity. The preceding winter had been damp and rainy, and the wind blowing almost constantly from the south, and the spring had set in with sultry heat. The epidemic constitution had been remarkable for several months for the frequency of the eruptive fevers; intermittents, too, had been very common.

The disease prevailed especially in damp and marshy situations, and was particularly severe along the borders of the Lisone, a river of Angouleme, which overflows its banks, and renders the neighbouring country swampy. The persons attacked by the epidemic were almost all between twenty and forty-five years old: youth and old age appearing to be alike exempt from its seizure. No satisfactory statements appear with reference to the mortality occasioned by the disease; it appears, however, to have differed much in different districts, and the death of several individuals was attributed by the physicians to the effects of panic rather than of the malady.

In its milder form the disease was ushered in by sense of pain and heaviness in the head, and cardialgia, which having lasted for some hours, or even for a whole day, were followed by sweat. It happened, however, sometimes that the sweat broke out suddenly, without any premonitory symptom, while persons were engaged in their ordinary occupations; or that, waking in the night, they found themselves bathed in perspiration. A sense of tingling in the skin usually preceded the sweat; after it had broken out the patient had fever, with redness of the face, singing in the ears, great sensibility of the eyes, tenderness of the abdomen, hemorrhage from the nose, nausea, loss of appetite, and vomiting, but without thirst.

After the lapse of twenty-four or seventy-two hours the eruption made its appearance, and usually announced the termination of the disease. The eruption was most abundant about the arms, chest, face, and precordia; it usually consisted of sudamina, which sometimes were too minute to be distinguished by the naked eye; but sometimes it had more the appearance of eczema, or showed itself in large red patches like scarlatina, with interspaces where the skin retained its natural appearance. The disease usually ran its course in from six to ten days. The shortest interval between the premonitory symptoms and the outbreak of the eruption was three days, the longest six days, and desquamation followed after about three or four days more.

In those cases where the affection assumed a malignant character, it appeared suddenly and after brief but violent premonitory symptoms, abundant fetid sweats broke out over the whole body. The tongue soon became dry and tremulous, the face sunken, the eye haggard, the countenance had a leaden hue; there was delirium occasionally, with syncope, automatic movements, carphologia, and death followed in seven or eight hours.

In the milder cases very little medical treatment was adopted beyond the enjoining abstinence and rest, and taking care that the patient did not expose himself imprudently to the cold. Whenever the fever showed signs of remission or intermission, sulphate of quinine was employed, and symptoms of malignancy were combated by musk, æther, and similar remedies.

This epidemic appeared in almost all points to have resembled that which prevailed in the department De l'Oise; no notable difference of opinion arose among the medical practitioners with reference to its treatment, and no important addition has been made by the reports of the physicians who witnessed it, to our knowledge, of the disease.

*Bulletin de l'Académie Royale, Nov.* 30, 1841.

---

*Memoir on Softening of the Brain.* By M. DURAND FARDEL.

THE object of this paper is to illustrate the inflammatory nature of the disease. Several observations are detailed in which acute *ramollissement* occurred; and in all there existed redness of the brain, as well as softness of its tissue. This redness was in most instances produced by a partial and well-marked injection of vessels; in others there was sanguineous infiltration, as well as vascular injection. The blood was in general evidently derived from the rupture of vessels. and though it existed in a few cases unaccompanied by any vascular injection, analogy would still lead us to refer it to the same cause. There are indeed but three sources from which hemorrhage into the tissue of an organ could be derived. It must either result from some alteration in the blood, in which case hemorrhage ought to occur simultaneously into the tissue of several organs; or it may be produced by some alteration in the coats of the vessels—an hypothesis in this case purely gratuitous, and opposed by the fact that ossification of the arteries of the cranium did not exist in any of the eight observations recorded in the paper. Lastly, cerebral congestion and consequent rupture of vessels would account for the occurrence of the redness which exists at the commencement of softening of the brain, and few will deny that softening of an organ consequent on cerebral congestion is the result of inflammatory action.

When death does not take place during the first stage of ramollissement, the disease passes into the chronic state, and changes in the cerebral structure occur in which three different stages may be noticed.

The first stage is characterized by the disappearance of the redness, without any other alteration in the condition of the softened brain. It is to the disease at this period that the description applies which most writers have given of softening of the brain.

In the second period, the ramollissement undergoes very different modifications, according to the part of the brain which it involves. In the cortical substance of the convolutions it assumes the form of yellow membraniform patches; but in the rest of the brain the softened parts become attenuated, the cellular texture of the cerebral substance becomes exposed and infiltrated with a turbid whitish liquid, which appears to consist of the nervous pulp liquefied. To this condition the author applies the name of cellular infiltration.

In the third period, the softened cerebral substance begins to disappear, and real ulcerations form on the surface of the brain, while in its interior the loss of substance gives rise to excavations which must not be confounded with the traces left by the cerebral hemorrhage.

It is difficult to state anything positively as to the time occupied by these changes. Sometimes they occur with great rapidity, while in other instances their advance is extremely slow—so slow, indeed, that it would almost appear as though the disease might remain stationary for an indefinite length of time in any one of its stages. The advance of the changes of the brain is usually indicated by a corresponding progress in the gravity of the symptoms, while the arrest of the disease is accompanied by a corresponding improvement in the patient's condition.

There is a further distinction necessary to be made between the cases in which the ramollissement remains circumscribed to its original limits and those in which the disease has extended to surrounding parts, so as to present the appearance of a brain, different parts of which are in different stages of ramollissement. These latter cases have afforded M. Fardel the opportunity of ascertaining the various stages of softening of the brain which form the subject of this memoir.

The cases related by M. Fardel, and the minute anatomical details into which he enters do not admit of abstract. The memoir, however, is highly interesting, and will well repay a careful perusal.

*Archives Générales de Médecine. Jan. and Feb.* 1842.

---

*Researches into the Physical Causes of Metallic Tinkling, or Amphoric Ronchus.* By M. de Castelnau, House-Surgeon (interne) of the Hospitals.

The author commences this paper by reviewing the different theories which have been suggested to explain this phenomenon, and pronounces them all more or less unsatisfactory. The hypothesis most usually adopted, and which attributes the sound to the bursting of an air-bubble on the surface of the fluid effused into the pleural cavity, is regarded by him as equally defective with the others. Experiments which he details have led him to the conclusion, that the formation of bubbles of air at the surface of an effusion is almost impossible, even in those cases where the perforation of the pleura is below the level of the fluid, while its occurrence is altogether out of the question when there exists perforations of the pleura above the level of the effusion.

The occurrence of pulmonary fistula, however, above the level of an effusion is by no means unusual; it is even stated by M. Raciborski to be the case in by far the greater number of instances. Laennec, too, had observed that, after the operation for empyema, in which the puncture is made above the level of the fluid, metallic tinkling is frequently heard; while if the wound had been made too large the respiration acquires an amphoric sound. This phenomenon can be explained only by supposing metallic tinkling to be a variety of the amphoric sound; and M. C.'s experiments on the dead subject have convinced him that such is really the case. He further deduces from them the following conclusions:

1. That the physical conditions essential to the production of metallic tinkling are: *a*, The existence of a tolerably large cavity, containing air, either with or without fluid; *b*, The communication of the external air with this cavity; *c*, The production of sonorous vibrations in the channels by which this communication is established.

2. The causes which give rise to these vibrations are identical with those which produce moist sounds in general.

3. Metallic tinkling may be called an amphoric ronchus, with as much propriety as the term amphoric may be applied to the respiration, voice, or cough.

4. Those cases, if indeed any such exist, in which metallic tinkling occurs independent of the above-mentioned conditions, are exceptions to the rule, as are also the theories advanced in explanation of them.

In confirmation of these views, a case is related in which metallic tinkling was heard, and the respiration, voice, and cough had an amphoric sound in a phthisical patient. After death the left lung was found to be occupied by two very large cavities, which had destroyed the greater part of its substance. A septum, only three or four lines thick, separated the two cavities from each other. The superior was empty; the other contained about four ounces of broken-down tuberculous matter, in a semi-fluid rather than a liquid state, and the openings of the bronchi into the cavity were all, with the exception of two, situated above the level of the softened tubercle. Similar phenomena were observed in the case of a man in whom fracture of the ribs and clavicle was followed by subcutaneous emphysema and pneumothorax. The metallic tinkling and amphoric respiration disappeared gradually as the man advanced towards convalescence; and there was no reason, at any period of his illness, to suppose the existence of fluid in the cavity of the pleura. He likewise alludes to a third case, in which a wound in the chest with a knife, though unaccompanied with subcutaneous emphysema, or even with hæmoptysis, was followed by metallic tinkling and amphoric sounds.

In a second paper on the same subject, two other cases are adduced in confirmation of the author's views.

*Archives Génèrales de Médecine. Oct. et Nov.* 1841.

---

*Analysis of the Urine in Scarlatina.* By Dr. S. F. Simon, of Berlin

The urine in scarlatina almost always presents the characters of the urine of inflammation, being scanty, of a deep red colour, strongly acid, and often of a higher specific gravity than in health. In the stage of desquamation it becomes more copious, but retains its dark colour, and often contains albumen.

Dr. Simon analysed the urine of a boy aged five years, suffering under scarlet fever, with considerable affection of the sensorium and putrid odour from mouth and nose. The urine had a dark yellow colour, was slightly acid when first voided, but soon became alkaline on standing, and then threw down a copious white sediment. Under the microscope, this sediment was seen to be composed of large opaque globules, and a fine granular deposit, with mucous globules and a few crystals of phosphate of ammonia and magnesia. It was almost completely dissolved when heated, and small rhomboid crystals of lithic acid appeared on treating it with hydrochloric acid. The amorphous residuum consisted of lithate of soda and lithate of ammonia. During desquamation the urine still continued to become very speedily alkalescent, and still threw down the white sediment, but without becoming perfectly clear. No epithelium scales could be discovered in this sediment, but large masses of epithelium cells existed in the turbid urine which covered the sediment, and some of the cells seemed to have been slightly acted on by the alkalescence of the urine. The above fact shows that the process of desquamation extends to the lining of the bladder.

The urine of this patient had a specific gravity of 1022; 1000 parts contained 56·7 of solid matter, of which 19·3 were urea, and 1·64 lithic acid, which was in a state of combination with bases in the urine.

*Allgemeine medicinische central-Zeitung. Feb.* 5, 1842.

---

*Researches into the real Nature of Œdema of the Glottis.* By M. Bricheteau, Physician to the Hospital Necker.

The object of this paper is to disprove the existence of œdema of the glottis as a distinct disease, unconnected with other affections of the larynx. M. Bricheteau criticises the facts adduced in Bayle's paper on the subject, and laments their incompleteness, as well as that of the cases which are found in the thesis of M. Thuillier. M. Bouillaud published three observations in the year 1825 tending to prove that this disease is in reality an inflammation of the larynx, pharynx, and surrounding parts. The remarks contained in the prize essay of MM. Trousseau and Belloc favour the same conclusion, which is further strengthened by the three cases detailed by the present author. Two of these cases terminated in recovery, but in the third the patient sank after the performance of tracheotomy. Some disease of the larynx existed in all three instances, to which œdema of the glottis succeeded.

This œdema, which, strictly speaking, affects the lips of the glottis not the glottis itself, is always a very serious affection, on account of the seat which it occupies. It cannot, however, be regarded as an idiopathic disease, and is itself more frequently an inflammatory affection or a purulent infiltration into the part than a serous effusion. It supervenes in the course of various diseases of the larynx, and should not occupy a different place in works on pathology from œdema or tumefaction of a part produced by any morbid poison, or from the various symptoms which attend syphilis, or from ulcerations of the intestines in fever.

*Archives Générales de Médecine. Nov.* 1841.

---

*Account of a Case of Acute Glanders in the Human Subject.* By M. Bouillaud.

A youth aged seventeen years, a day labourer, was admitted into the Charité under the care of M. Velpeau. He had been ill for three weeks, from a swelling at the upper part of his chest, which was treated by the application of a few leeches, but without much benefit, and after being confined to bed at his own home he entered the hospital. After remaining for a fortnight under M. Velpeau's care, symptoms of erysipelas of the face came on, and the patient fell into a typhoid state, on which account, after having been bled by M. Velpeau's orders, he was transferred to the medical wards, and came under the care of M. Bouillaud on June 27, 1841.

There existed at that time some tumefaction of the face, the skin of the forehead was tense and glistening, and the eyelids were so much swollen as to close the eyes completely. The skin generally was very hot, the abdomen rather tympanitic, the face somewhat sunken. He was bled from the arm, and on the following day expressed himself as being slightly relieved, but there was greater depression of countenance; the pulse had risen from 120 to 132, and was soft. There existed great feeling of weakness, and the patient had been restless and delirious during the night; one eye was closed, the other partially open; the lips were dry; the patient expectorated a little viscid matter from the mouth, and a little blood was mixed with some expectoration derived from the posterior nares, but there was no running from the nose.

On the 29th delirium had become more constant, the prostration and typhoid symptoms had increased, the face continued swollen, and the eyes were closed. The delirium continued through the following day, and on the 30th the patient was evidently sinking, and died in the course of the day. His face, however, on that day presented an eruption, likewise evident on the limbs, the pustules of which were intermediate in size between those of ecthyma and variola, and destitute of any central depression. The eyelids were agglutinated by a purulent secretion; and, in addition to the tumefaction already noticed, there was a circumscribed swelling above the eyebrows, which presented numerous, confluent, livid pustules, in which ulceration was just commencing.

It was this eruption, precisely similar in its appearance to that which has been described as characteristic of glanders, that led M. Bouillaud to suspect the real nature of the affection, and induced him to examine the cavities of the nose and mouth after death with particular care. No peculiar excretion was found in the mouth or nares, but the mucous membrane was universally red, and in the nares and back of the pharynx were very numerous grayish patches, produced either by ulcerations or by pustules which appeared to be the origin of ulcerations. The ulcerations were not deep, they varied in size from four to twelve millimetres, and between them small whitish pustules were interspersed. The pustules noticed during life on the surface of the body were flaccid after death, but contained pus of a yellowish colour, and several small purulent depôts were found beneath the skin of the right forearm. The particular eruption above the eyebrows had put on a gangrenous appearance.

The appearance of the pustular eruption during life, and the state of the membrane of the mouth and nares found after death, accord exactly with what are described as the characteristics of glanders. The absence of running from the nose might indeed throw some doubt upon the nature of the disease, but since it was ascertained after the patient's death that for six months he had been daily occupied with grooming a glandered horse, the case must be regarded as proving the possibility of glanders putting on a latent form, and arriving almost at its termination before it displays those symptoms which in the present state of our knowledge are regarded as diagnostic.

M. Bouillaud attaches considerable value to the pseudo-varioloid eruption described in the foregoing case, as characteristic of glanders, and alludes to three other recent instances in which it was observed.

*Bulletin de l'Académie Royale. December* 15, 1841.

In the same journal are two other cases of glanders in man, the former of which is described with considerable minuteness in the Archives Générales de Médecine for December, 1841, by Mr. Tardieu.

The first case is one of chronic glanders terminating in acute glanders. The subject of the observation, a man thirty-four years old, served as groom at the veterinary school of Alfort for three years, from 1834 to 1837, during which time he had daily to attend to glandered horses. About the end of this time he began to suffer from constant pain in the throat, he felt stuffed in the head, and blood came whenever he blew his nose. He consulted several medical men without relief; but having left Alfort, continued his occupation as a groom, and often had the care of glandered horses. About the middle of July 1841, an abscess on the left instep forced him to seek admission into the Charité. Immediately after he entered the hospital, a large fungoid ulceration was observed on the palate, which resisted a mercurial treatment and frequent cauterizations. He grew weaker, his constitution broke down, and on the 5th November the symptoms of acute glanders appeared, and proved fatal in five days.

On a post-mortem examination all the ordinary consequences of acute pustular glanders were found as well in the nasal fossæ as in the viscera. There existed, however, besides traces of old disease, 1, In the nose; 2, In the palate, where beneath the ulcerated mucous membrane the bone was found rough and pierced with holes, as though it had been ulcerated; 3, In the air-passages, whose internal surface, from the upper extremity of the epiglottis to the division of the bronchi, was but one vast cicatrix, which had evidently existed for some time. These lesions have often been observed in horses, and are of a character not to be mistaken; but this is the first instance in which cicatrices in the trachea, the result of farcy or glanders, have been observed in the human subject.

The second case is that detailed in our last No., p. 235.

---

*Researches on Saccharine Diabetes.* By Dr. BOUCHARDAT.

THIS is the second of two papers on the subject, read by M. Bouchardat at the Academy of Sciences. In his former essay he showed that the quantity of sugar in diabetic urine is in direct proportion to the bread or feculent substances which the patient takes for food. He further ascertained that the water which persons suffering under diabetes take in such abundance is not usually more than is necessary to effect completely, by means of the diastase, the transformation of the fecula into sugar.

From these observations he had deduced the conclusion that, for the cure of diabetes nothing more is requisite than to withhold as far as possible all fluids, or articles of diet containing sugar or fecula; and the present paper contains the results of some cases in which this plan was adopted.

M. Bouchardat found it impossible to withhold farinaceous food and fluids as completely as he wished, and consequently his success was but temporary. The nutritive properties of gluten having recently become more known by the researches of the *commission de la gelatine,* he next endeavoured to form from it an aliment which might supply the place of bread. In this he so far succeeded as to have formed an exceedingly palatable substitute for bread, with the addition of only a fifth of farina. The beneficial effects of this substitute for bread are shown in two tables; from which it appears, that when common bread and that made from gluten were given on alternate days, the employment of the latter was always accompanied with an extremely marked diminution both in the quantity of urine and in the amount of sugar it contained. The next indication would be to promote the perspiration, and to restore to a healthy character the secretion of the diseased mucous membrane of the stomach. M. Bouchardat insists on flannel being worn next the skin, and regards it, with the employment of opiates and preparations of ammonia, as the means best calculated to promote diaphoresis.

The histories of four patients who were submitted to this treatment are detailed. Two received but slight benefit; a third was cured so completely that all traces of sugar disappeared from his urine, which presented in all respects the characters of the healthy secretion; and so small a proportion of sugar is contained in the urine of the fourth patient, that M. Bouchardat confidently anticipates his perfect recovery. It is worthy of observation that the treatment was not altogether identical in the four cases; for the first two did not wear flannel garments next their skin, and the employment of carbonate of ammonia in those instances was followed after two days by a change in the urine from acid to alkaline, together with a slight increase in the quantity of sugar. In the other two cases, the thickness of the flannel garment was always increased whenever the sugar in the urine became more abundant; and, notwithstanding the administration of the carbonate of ammonia, the urine of these two patients continued acid throughout.

*Comptes Rendus de l'Académie. Nov.* 15, 1841.

---

*Case of Larvæ of Insects (Musca Stabulans) passed by Stool.* By Professor Hoppe.

A girl of thirteen, of weak constitution, and whose father was in a high degree epileptic, was suddenly attacked with convulsions in November, 1839, which again returned in March, 1840. Upon the administration of vermifuge powders with large doses of ol. ricini, a great number of larvæ of the above-named insects were passed by stool. Occasional convulsive attacks recurred during the succeeding summer, and always subsided when similar evacuations were obtained by the same remedies. The convulsive paroxysms seldom lasted above two minutes at a time, and occurred only in the mornings before ten, A. M. In December last the patient had been for four months in the enjoyment of the most perfect health. The convulsive attacks were occasionally preceded by a peculiar imbecility of mind, so that, in endeavouring to leave the room, she was often totally unable to discover the door. Professor Hoppe, who relates this singular case, believes the larvæ to have been swallowed in fruit or vegetables during the preceding autumn.

*Bibl. for Læger. December,* 1840.

---

*On Typhus in the Royal Frederick's Hospital at Copenhagen.* By Professor Bang.

Professor Bang presents us with a statistical view of 890 cases of true typhoid fever, in the Royal Frederick's Hospital at Copenhagen, in the years 1837-8-9. The number of cases of typhus admitted in 1837 was 214; in 1838, 472; in 1839, 204. The average mortality of these three years was 216 in 890, or 1 in 4, nearly.

The fever began with common febrile symptoms, seldom exhibiting at the very commencement the malignant typhoid character. The pulse was generally full and strong, the bowels open; occasionally there appeared an exanthematous eruption (typhöse exanthem), but petechiæ were extremely rare. The typhoid exanthema was more or less of a red colour, and irregularly distributed over the body.

The disease showed itself under four different forms; i.e. cerebral typhus, pectoral typhus, abdominal typhus, vesical typhus.

| | | | |
|---|---|---|---|
| In cerebral typhus the mortality was | | | 1 in 3·8. |
| In pectoral typhus | ,, | ,, | 1 in 3·1. |
| In abdominal typhus | ,, | ,, | 1 in 5·3. |
| In vesical typhus | ,, | ,, | 1 in 2·5. |

The cerebral, abdominal, and pectoral forms presented nothing remarkable, but vesical typhus has hitherto been but little noticed. This form prevailed chiefly in 1838 and 1839, attacking 28 individuals, 11 of whom died. The age of those attacked was, from 16 to 20—6, 2 of whom died; 20 to 30—5 attacked, 1 died; 30 to 40—3 attacked, all recovered; 40 to 50—8 attacked, 5 died; 50 to 60—6 attacked, 3 died. In all the 28 cases pain was felt on pressure above the pubes; in 14 pain was complained of there without pressure; in 24 cases ischuria at first was present, which was replaced in 23 cases by enuresis; in 26 cases the urine let fall a muco-purulent and occasionally a sanguineous deposit, which was viscid, and adhered firmly to the sides of the vessel. In all these cases a putrid smell was exhaled from the urine, but in only 20 did it present any ammoniacal odour. It occurred 6 times with cerebral typhus, once with pectoral typhus, and 21 times in abdominal typhus. The typhoid exanthema appeared in 12 out of the 28 cases, and the fever always partook more or less of the hectic character. The vesical complication did not appear at the commencement of the disorder, but generally between the second and third weeks, and was to be recognized particularly by ischuria, which, however, was often concealed by the concomitant enuresis, by pain above the pubes, and shortly after by the peculiar appearances in the urine above described. The results of dissection in 9 cases of vesical typhus are exhibited in the following table.

| | |
|---|---|
| Inflammation of the entire bladder in . . | 3 |
| ditto of fundus of ditto . . | 1 |
| Ulcerations in neck of ditto . . | 3 |
| ditto in fundus of ditto . . | 1 |
| Extravasation of the mucous membrane . | 3 |
| Petechiæ . . . . . . . | 2 |
| Small ossifications . . . . . | 1 |

The vesical ulcerations resembled closely those observed in the intestinal canal in abdominal typhus.

The treatment of the vesical form of typhus consisted in the application of leeches above the pubes, followed by warm fomentations, which, however seldom relieved the pain, until Professor Bang administered a mixture of sulphuric acid and opium, which combination he found to be the most efficacious in this severe form of typhus.

In all the above-mentioned varieties or forms of typhus, he strenuously recommends the employment of active purgatives, often administering calomel in doses of from 15 to 25 grains. He believes the diminution in the rate of mortality of last year, viz. 115 deaths in 724, entirely to arise from the active purgative treatment he has recently adopted in typhus.

*Bibl. for Læger. August*, 1840.

---

*On the Utility of an Ointment of the Oxide of Zinc in the Treatment of Eczema, Impetigo, and Ecthyma.* By M. MARTIN SOLON.

THE formula of M. Solon consists of,

Lard, 30 grains.
Oxide of zinc, from 1 to 3 grammes. (15 to 45 grs.)
Mix.

To be applied to the parts night and morning.

In eczema this ointment allays the itching, diminishes the redness and oozing from the surface, and greatly expedites the cure.

In that form of impetigo to which Alibert gave the name of melitagra, it is very useful after the scabs have once fallen off. It acts by diminishing the secretion of the skin, and preventing the formation of new pustules. It likewise promotes the desiccation of the pustules of ecthyma.

M. Solon does not recommend it as a specific in these affections, but merely as a valuable topical application.

*Bulletin Général de Thérapeutique. Novembre*, 1841.

